# Symbiotic cornucopia of the monophagous planthopper *Ommatidiotus dissimilis* (Fallén, 1806) (Hemiptera: Fulgoromorpha: Caliscelidae)

**DOI:** 10.1007/s00709-018-1234-0

**Published:** 2018-03-07

**Authors:** Anna Michalik, Jacek Szwedo, Adam Stroiński, Dariusz Świerczewski, Teresa Szklarzewicz

**Affiliations:** 1 Department of Developmental Biology and Morphology of Invertebrates, Institute of Zoology and Biomedical Research, Jagiellonian University, Gronostajowa 9, 30-387 Kraków, Poland; 2 Department of Invertebrate Zoology and Parasitology, University of Gdańsk, Wita Stwosza 59, 80-309 Gdańsk, Poland; 3 Museum and Institute of Zoology, Polish Academy of Sciences, Wilcza 64, 00-679 Warszawa, Poland; 4 Department of Zoology and Animal Ecology, Jan Długosz University, Armii Krajowej 13/15, 42-201 Częstochowa, Poland

**Keywords:** Symbiotic microorganisms, *Sulcia*, *Vidania*, *Sodalis*, *Wolbachia*, *Rickettsia*

## Abstract

In contrast to Cicadomorpha, in which numerous symbiotic bacteria have been identified and characterized, the symbionts of fulgoromorphans are poorly known. Here, we present the results of histological, ultrastructural, and molecular analyses of the symbiotic system of the planthopper *Ommatidiotus dissimilis*. Amplification, cloning, and sequencing of bacterial 16S RNA genes have revealed that *O. dissimilis* is host to five types of bacteria. Apart from bacteria *Sulcia* and *Vidania*, which are regarded as ancestral symbionts of Fulgoromorpha, three additional types of bacteria belonging to the genera *Sodalis*, *Wolbachia*, and *Rickettsia* have been detected. Histological and ultrastructural investigations have shown that bacteria *Sulcia*, *Vidania*, and *Sodalis* house separate bacteriocytes, whereas bacteria *Wolbachia* and *Rickettsia* are dispersed within various insect tissue. Additionally, bacteria belonging to the genus *Vidania* occupy the bacteriome localized in the lumen of the hindgut. Both molecular and microscopic analyses have revealed that all the symbionts are transovarially transmitted between generations.

## Introduction

In the co-existence of insects and plants which has lasted over 400 million years, both of them have established different types of relationships with microbial associates, which have had a significant influence on plant–insect interactions. Nutritional symbiosis between insects and microorganisms (bacteria and/or yeast-like microorganisms) is one of the most interesting aspects of inter-species interactions. The role of symbionts is similar in different insect groups—they usually supplement their unbalanced diet with essential nutrients (amino acids in sap-feeding insects, vitamins and cofactors in hematophagous ones) (Dale and Moran 2006; Moran et al. 2008; Douglas 2016; Giron et al. 2017).

Being one of the Big Five insect orders, Hemiptera make up an unbelievably diversified and successive group, inhabiting all terrestrial and some marine habitats (Szwedo 2018). Hemiptera are characterized by a great diversity of symbiotic microorganisms which is a result of both multiple, independent acquisitions as well as symbiont replacement during their evolution (Bennett and Moran 2013, 2015; Koga et al. 2013; Toenshoff et al. 2013). It is believed that the beginning of the symbiosis between Euhemiptera and microorganisms took place over 270 mln years ago when the ancestors of Fulgoromorpha and Cicadomorpha were infected by two different bacteria (one from the phylum Bacteroidetes and another one from the Proteobacteria phylum, Betaproteobacteria class) (Moran et al. 2005; Bennett and Moran 2013, 2015).

In comparison with other hemipteran groups, there is still an insufficient amount of knowledge concerning the symbiotic microorganisms associated with planthoppers. Existing literature on the subject indicates that symbiotic systems of planthoppers are more complex than symbiotic systems observed in other hemipterans (Buchner 1925, 1965; Müller 1940a, b; Bressan et al. 2009; Michalik et al. 2009; Gonella et al. 2011; Urban and Cryan 2012; Bressan and Mulligan 2013). The first existing morphological studies on symbionts of Fulgoromorpha were carried out by Müller (1940a, b) and Buchner (1925). Based on paraffin sections, they revealed that planthoppers are usually host to two types of symbiotic microorganisms, which were then designated as *a*-symbionts and *x*-symbionts. Some of them, however, may possess a third type called *f*- or *m*-symbiont. More recently, Urban and Cryan (2012) have indicated that the obligatory symbionts in Fulgoromorpha belong to the species “*Candidatus* Sulcia muellerii” (hereafter referred to as *Sulcia*) (i.e., *a*-symbiont sensu Buchner and Müller) and “*Candidatus* Vidania fulgoroidea” (hereafter referred to as *Vidania*) (i.e., *x*-symbiont sensu Buchner and Müller) and co-evolve with their hosts. Bressan et al. (2009, 2013) and Gonella et al. (2011) identified the gammaproteobacterium *Purcelliella pentastirinorum* in Cixiidae planthoppers. Additionally, in some members of the families Delphacidae and Flatidae, yeast-like symbionts have been found (Noda et al. 1995; Cheng and Hou 2001; Xet-Mull et al. 2004; Michalik et al. 2009; Tang et al. 2010; Cao et al. 2015). Based on the results of molecular studies conducted by Urban and Cryan (2012) which revealed the occurrence of *Sulcia* and *Vidania* only in about 40% of the species examined, Bennett and Moran (2013) hypothesized that *Sulcia* and *Vidania* are the ancestral symbionts of planthoppers, which have been lost and replaced by other symbiotic microorganisms in some families. So far, little is known about the frequency of symbiont replacement, their distribution in the bodies of planthopper, and mode of intergeneration transmission.

The insect studied, *Ommatidiotus dissimilis* (Fallén, 1806), belongs to the small but diverse planthopper family of Caliscelidae Amyot et Audinet-Serville, 1843. *O. dissimilis* is the Euro-Siberian species which inhabits wet and marshy habitats and monophagously feeds on hare’s-tail cottongrass *Eriophorum vaginatum.*

The aim of this work was, therefore, to describe the complexity of the symbiotic system of the planthopper *O. dissimilis* and verify whether it possesses *Sulcia* and *Vidania* symbionts, as well as whether these ancestral bacteria are associated with additional symbiotic microorganisms, or if have been lost and replaced by “new” symbionts. To address these questions, detailed investigations of microorganisms associated with *O. dissimilis* using histological, ultrastructural as well as molecular methods were performed.

## Material and methods

### Host insect—*Ommatidiotus dissimilis*

The females of *O. dissimilis* were collected near Częstochowa—“Bagno w Korzonku” Natura 2000 site; Korzonek, community Konopiska, Upper Silesia from the hare’s-tail cottongrass *Eriophorum vaginatum* L. (Cyperaceae, Poales), between the years 2014 and 2016. The specimens were preserved in 100% ethanol (for the molecular studies) and 2.5% glutaraldehyde in 0.1 M phosphate buffer (pH 7.4) (for the histological studies) and then stored in a refrigerator (4 °C). Taxonomically, *O. dissimilis* belongs to the tribe Ommatidiotini (Fieber, 1875) of the subfamily Ommatidiotinae (Fieber, 1875).

### Light (LM) and electron microscopy (TEM)

The dissected abdomens of 20 adult females, destined for detailed histological and ultrastructural analysis, were fixed in 2.5% glutaraldehyde in 0.1 M phosphate buffer (pH 7.4), rinsed in the buffer with the sucrose (5.8 g/100 ml), postfixed in buffered 1% osmium tetroxide, dehydrated in an ethanol series (30, 50, 70, 90, 100%) and acetone, and finally embedded in epoxy resin Epon 812 (Serva, Germany). Semithin sections (1 μm thick) were stained with 1% methylene blue in 1% borax and photographed using the Nikon Eclipse 80i light microscope. The ultrathin sections (90 nm thick) were doubly contrasted with uranyl acetate and lead citrate and then examined and photographed under a Jeol JEM 2100, at 80 kV transmission electron microscope.

### Molecular analyses

The dissected abdomens of the adult females of *O. dissimilis* were fixed in 100% ethanol. Additionally, isolated, mature oocytes were used for the molecular analysis. DNA was extracted separately from 5 individuals and 10 oocytes using the Sherlock AX DNA extraction kit (A&A Biotechnology) following manufacturer protocol and subsequently stored at 4 °C for further analyses.

The 1.5-kb fragment of bacterial 16S RNA gene sequence was amplified using universal, eubacterial primers: 10F and 1507R (Sandström et al. 2001) under the following conditions: an initial denaturation step at 94 °C for 3 min, followed by 33 cycles at 94 °C for 30 s, 55 °C for 40 s, 70 °C for 1 min 40 s, and a final extension step of 5 min at 72 °C. The PCR product was made visible by electrophoresis in 1.5% agarose gel stained with Midori Green (Nippon Genetics Europe) and following this, the appropriate band was cut off and purified using the Gel-out purification kit (A&A Biotechnology). The purified PCR product was cloned to the pJET 1.2/blunt plasmid vector using Clone JET PCR Cloning Kit (Thermo Scientific). The ligated mixture was then transformed into competent *Escherichia coli* TOP10F cells, which were subsequently prepared using the *E. coli* Transformer Kit (A&A Biotechnology). After 16 h, the occurrence of bacterial 16S RNA genes was confirmed by diagnostic PCR reactions with the primers 10F and 1507R. To determine the diversity of bacterial microorganisms occurring in the body of *O. dissimilis*, 30 positive colonies were subjected to restrictive analysis using a *MspI* restrictive enzyme. The plasmids from the selected colonies were isolated using a Plasmid Mini AX kit (A&A Biotechnology) and then the representatives of each RFLP genotype were sequenced. The cloning step was repeated five times (using DNA isolated separately from five individuals). In order to determine the systematic affinity of the symbionts, the sequences obtained were compared with other 16S RNA gene sequences deposited in the GenBank database using BLAST.

Diagnostic PCR reactions were performed under the following conditions: an initial denaturation step was performed at 94 °C for 3 min, followed by 35 cycles at 94 °C for 30 s, Tm for 40 s, 70 °C for 1 min 30 s, and a final extension step of 5 min at 72 °C. Primers specific for symbiotic bacteria used in the diagnostic PCR reactions have been summarized in the Table 1.

**Table 1:** Primers and fluorochrome-labeled probes used in this study

Purpose	Primer name	Primer sequence (3′-5′)	Target gene	Annealing temperature	Source
Cloning of bacterial genes	10F	AGTTTGATCATGGCTCAGATTG	16S rRNA gene of Eubacteria	55 °C	Sandström et al. (2001)
	1507R	GTTACGACTTCACCCCAG			
Diagnostic PCR	16SA1	AGAGTTGATCMTGGCTCAG	16S rRNA gene of *Sodalis*-like bacteria	54 °C	Fukatsu and Nikoh (1998); Koga et al. (2013)
	Sod1248R	TCCGCTGACTCTCGCGAGAT			
	10CFB	AGAGTTTGATCATGGCTCAGGATG	16S rRNA gene of *Sulcia*	54 °C	Moran et al. (2005)
	CFB1515R	GTACGGCTACCTTGTTACGACTTAG			
	VidF	ATTGGACAATGAGCGAAAGC	16S rRNA gene of *Vidania*	54 °C	This study
	VidR	GCGGTGTGTACAAGACCTGA			
	WspF	TGGTCCAATAAGTGAGAGAAAC	16S rRNA gene of *Wolbachia*	55 °C	Zhou et al. (1998)
	WspR	AAAAATTAAACGCTACTCCA			
	NcRic_16S/f1	TGACGGTACCTGACCAAGA	16S rRNA gene of *Rickettsia*	52 °C	Noda et al. (2012)
	NcRic_16S/r1	AAGGGATACATCTCTGCTT			
FISH	Sod1248R	Cy3-TCCGCTGACTCTCGGGAGAT	16S rRNA gene of *Sodalis*-like bacteria	Not applicable	Koga et al. (2013)
	BET940R	Cy5-TTAATCCACATCATCCACCG	16S rRNA gene of Betaproteobacteria	Not applicable	Demanèche et al. (2008)
	Sul664R	FITC-CCMCACATTCCAGYTACTCC	16S rRNA gene of *Sulcia*	Not applicable	Koga et al. (2013)

The nucleotide sequences obtained were deposited in the GenBank database under the accession numbers MG515259–MG515266.

### Fluorescent in situ hybridization

Fluorescent in situ hybridization (FISH) was conducted with symbiont-specific probes (see Table 1). Ten females preserved in 90% ethanol were rehydrated, fixed in 4% formaldehyde, and dehydrated through incubations in 80, 90, and 100% ethanol and acetone. Then material was embedded in resin Technovit 8100 and cut into sections. Hybridization was performed using a hybridization buffer containing the following: 1 ml 1 M Tris-HCl (pH 8.0), 9 ml 5 M NaCl, 25 μl 20% SDS, 15 ml 30% formamide, and about 15 ml of distilled water. The slides were incubated in 200 μl of hybridization solution (hybridization buffer + probes) overnight, at room temperature (Łukasik et al. 2017). Next, the slides were washed in PBS three times for 10 min, dried and covered with ProLong Gold Antifade Reagent (Life Technologies). The hybridized slides were then examined using a confocal laser scanning microscope Zeiss Axio Observer LSM 710. The FISH experiments were done four times. In each case, no-probes control experiments were performed.

### Phylogenetic analyses

The phylogenetic analyses were performed on the basis of the sequences of 16S RNA genes of symbionts of *O. dissimilis* and homologous sequences were downloaded from the GenBank database. The sequences were then edited using BioEdit Sequence Alignment Editor 5.0.9 (Hall 1999), whereas the alignments were generated using ClustalX 1.8 (Thompson et al. 1997). The base compositions of all of the genes analyzed were estimated using MEGA 7.0. software (Kumar et al. 2016). Phylogenetic analyses were conducted using MrBayes 3.2.2 software (Ronquist et al. 2012). In this analysis, four incrementally Metropolis coupling MCMC chains (three heated and one cold) were run for a total of ten million generations. The results of the Bayesian analysis were put into visual form using FigTree 1.4.0 software (Rambaut 2009).

## Results

### *Ommatidiotus dissimilis* is a host to five types of bacterial microorganisms

The bacteria residing in the body of *Ommatidiotus dissimilis* were identified as *Sulcia*, *Vidania*, and bacteria belonging to the genera *Sodalis*, *Wolbachia*, and *Rickettsia*. The 16S RNA gene sequences of *Sulcia* and *Vidania* symbionts were identical in each of the individuals examined; however, small differences between the 16S RNA genes of *Sodalis*-like symbionts (98–99% similarity) were observed.

A comparison of the sequences obtained with the data deposited in the GenBank has indicated that the 16S RNA genes of *Sulcia* had 96% similarity to the 16S RNA genes of *Sulcia* symbiont of other representatives of planthoppers such as *Neolieftinckana fuscata* and *Desudaba danae*, both members of family Fulgoridae. The 16S RNA genes of *Vidania* symbiont, however, exhibited the highest similarity with the *Vidania* symbiont of the planthoppers *Dictyssa* sp. (Tropiduchidae) and *Paracatonia* sp. (Achilidae). Interestingly, *Sodalis*-like symbionts of *O. dissimilis* had the closest matches with the free-living bacterium *Sodalis praecaptivus* (98% identity), *Sodalis*-allied symbiont of the shield bug *Picromerus lewisi* (Pentatomidae) (98% identity) and *Sodalis glossinidius* of tsetse fly *Glossina morsitans* (Glossinidae) (97% identity).

**Fig. 1:**
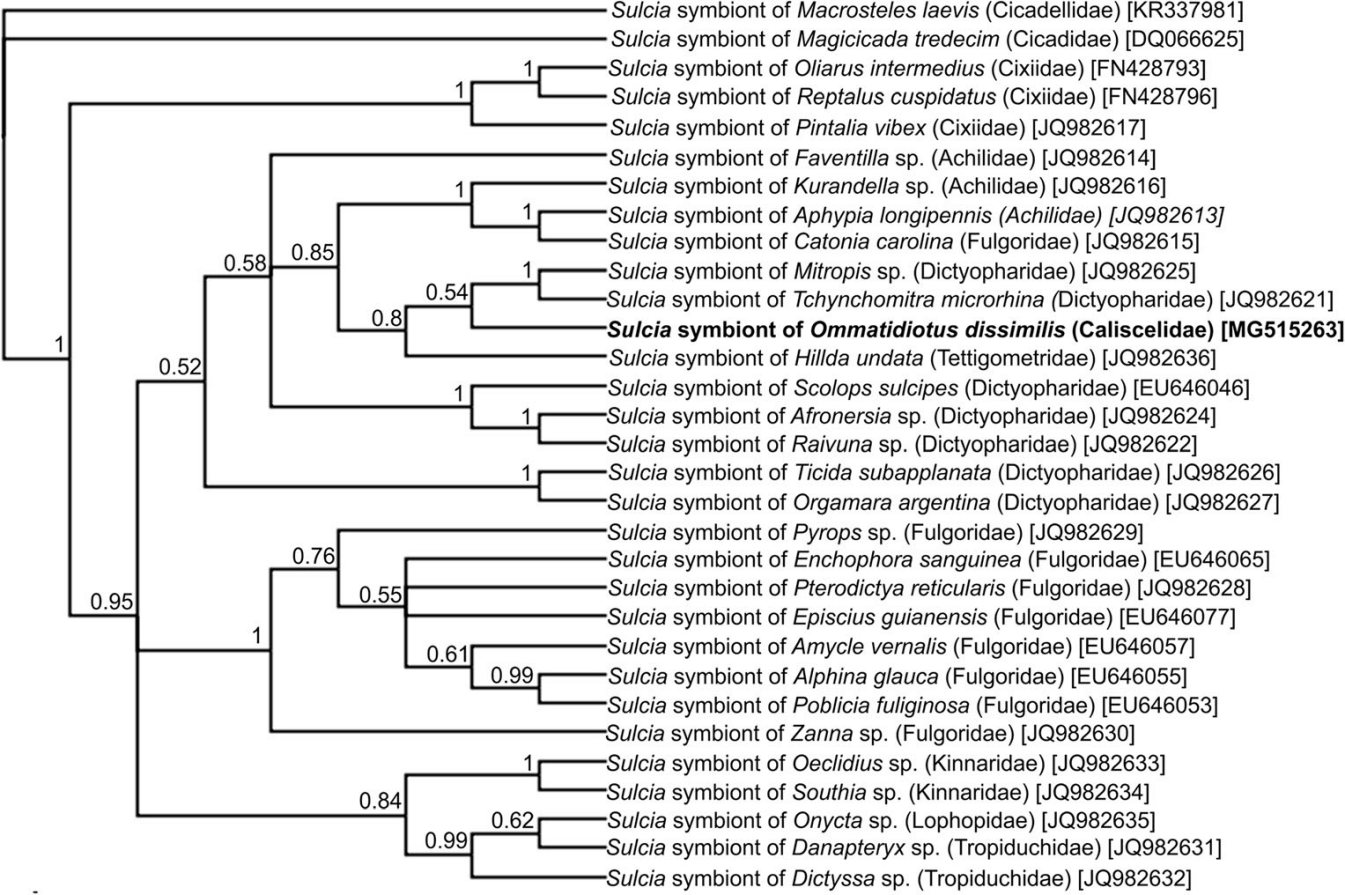
A cladogram showing the relationships of *Sulcia* symbionts of the examined leafhopper *Ommatidiotus dissimilis* and other representatives of Fulgoromorpha based on 16S RNA gene sequences. The numbers associated with the branches indicate the Bayesian posterior probabilities values. The accession numbers of the sequences used in the phylogenetic analysis have been put in brackets. For outgroups, *Sulcia* symbionts of *Magicicada septendecim* (Cicadidae) and *Macrosteles laevis* (Cicadellidae) were used

**Fig. 2:**
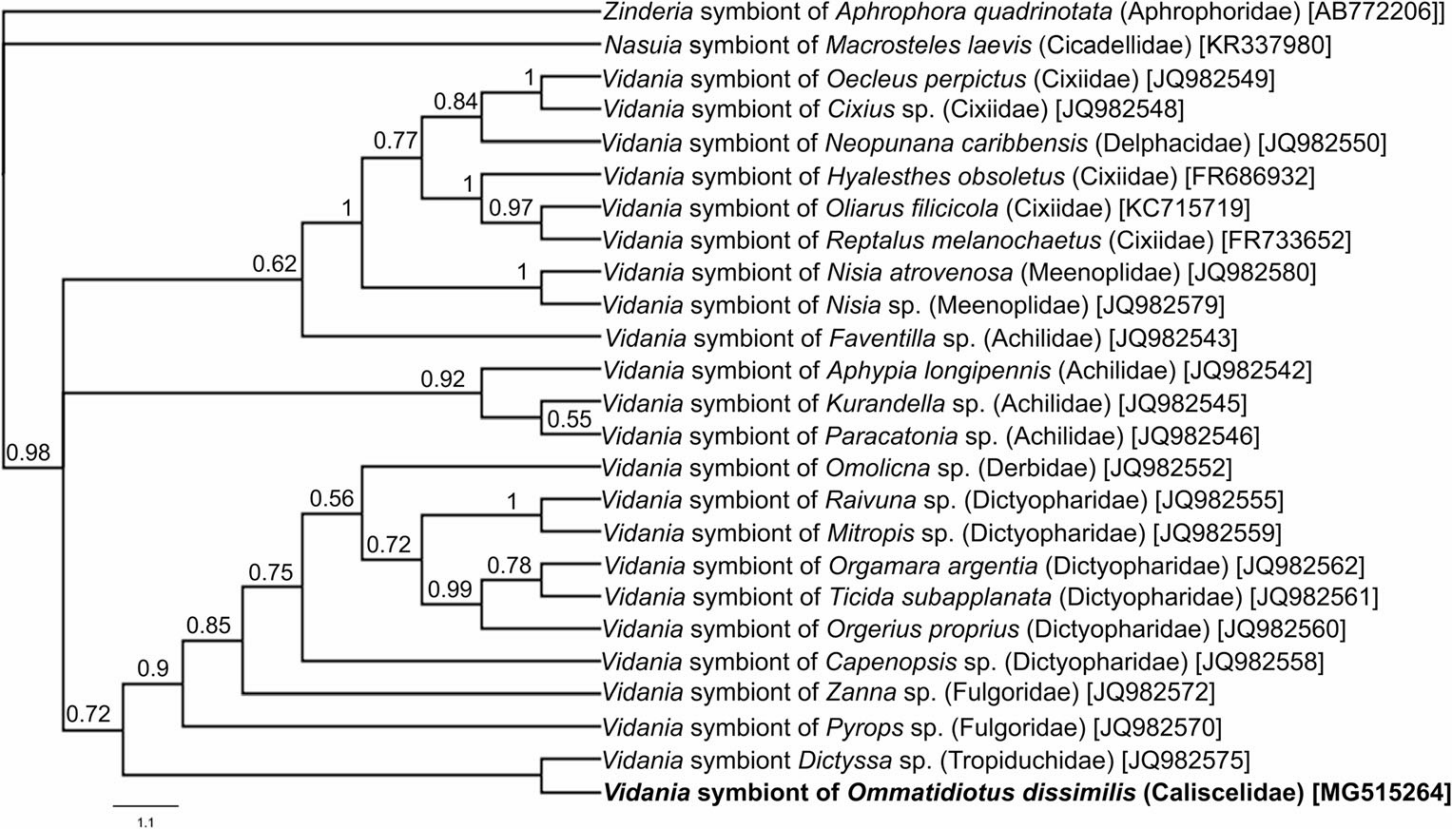
A cladogram showing the relationships of *Vidania* symbiont of the examined leafhopper *Ommatidiotus dissimilis* and other representatives of Fulgoromorpha based on 16S RNA gene sequences. The numbers associated with the branches indicate Bayesian posterior probabilities values. The accession numbers of the sequences used in the phylogenetic analysis have been put in brackets. For outgroups, *Nasuia* symbiont of *Macrosteles laevis* (Cicadellidae) and *Zinderia* symbiont of *Aphrophora quadrinotata* (Aphrophoridae) were used

Due to the fact that bacteria *Sulcia* and *Vidania* are regarded as ancestral symbionts of planthoppers (Bennett and Moran 2013), the phylogenetic analyses were performed on the basis of their 16S RNA gene sequences and the homologous sequences of other representatives of Fulgoromorpha. The results of the Bayesian analysis of *Sulcia* symbiont of Fulgoromorpha have revealed that the *Sulcia* symbiont of *O. dissimilis* is closely related to *Sulcia* isolated from *Mitropis* sp. and *Tchynchomitra microrhina*, both Dictyopharidae (Fig. 1). Bacteria *Vidania*, on the other hand, create a monophyletic clade with the *Vidania* symbiont of *Dictyssa* sp. (Tropiduchidae) (Fig. 2). The total length of the sequences which were subjected to phylogenetic analyses was 1322 bp, and 816 bp, whereas the base composition was as follows: 23.7% T, 18.5% C, 32% A, and 25.9% G and 27% T, 17.1% C, 32.6% A, and 23.3% G for *Sulcia* and *Vidania* symbionts, respectively.

The obtained sequences of 16S RNA genes of *Rickettsia* bacteria had 99% identity with the 16S RNA genes of *Rickettsia* from the leafhopper *Cicadella viridis* (Cicadelidae) and the American dog tick *Dermacentor variabilis* (Acari: Ixodidae), whereas the homologous sequences of *Wolbachia* displayed the highest similarity (99%) to the *Wolbachia* isolated from the planthopper *Sogatella furcifera* (Delphacidae) and spittlebug *Aphrophora quadrinotata* (Aphrophoridae)*.*

### *Sulcia*, *Vidania*, and *Sodalis*-like symbionts are localized in separate bacteriocytes, whereas *Wolbachia* and *Rickettsia* are dispersed within different tissue of *Ommatidiotus dissimilis*

**Fig. 3:**
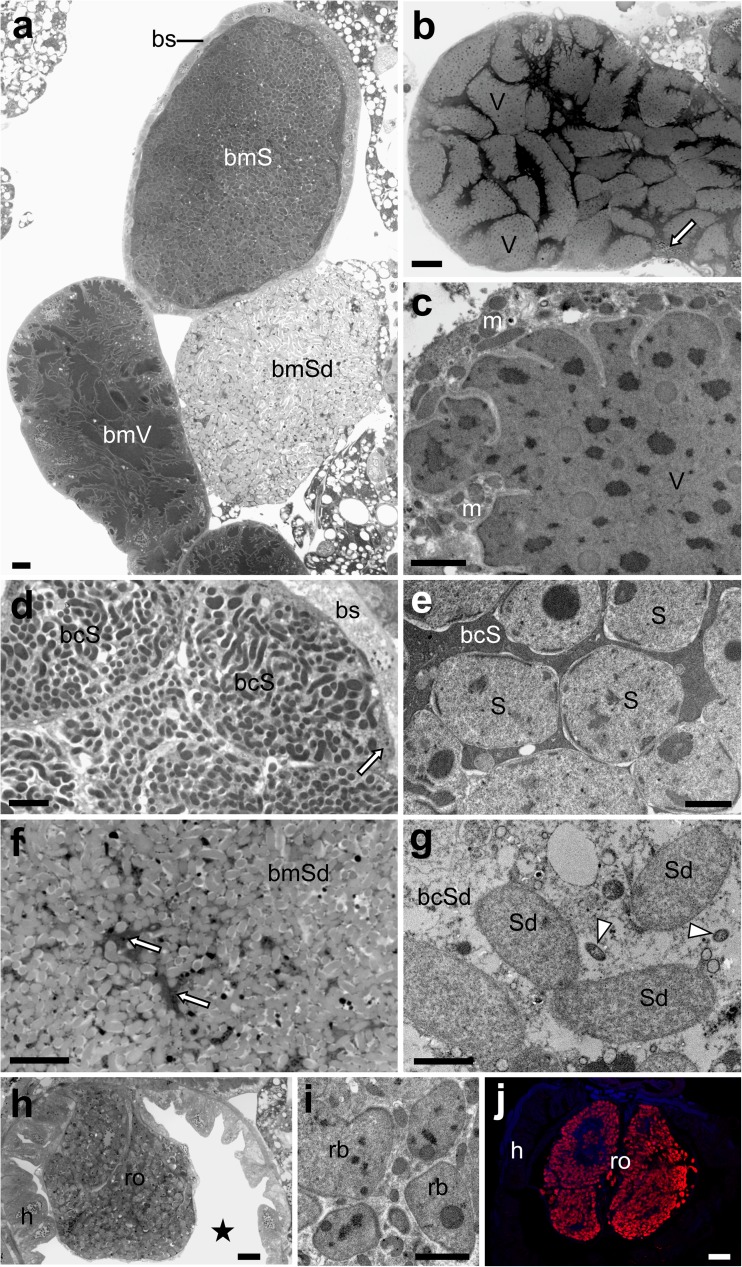
Distribution of symbiotic bacteria in the body of *Ommatidiotus dissimilis*. **a** The localization of the bacteriomes in the body cavity. Bacteriome with bacteria *Sulcia* (*bmS*); bacteriome with *Sodalis-like* bacteria (*bmSd*); bacteriome with bacteria *Vidania* (*bmV*); bacteriome sheath (*bs*). LM, scale bar = 20 μm. **b** Fragment of the bacteriome with bacteria *Vidania*. Bacterium *Vidania* (*V*); nucleus of the bacteriocyte (*white arrow*). LM, scale bar = 20 μm. **c** Fragment of the bacterium *Vidania* (*V*). Mitochondrium (*m*). TEM, scale bar = 2 μm. **d** Fragment of the bacteriome with bacteria *Sulcia*. Bacteriocyte with bacteria *Sulcia* (*bcS*); bacteriome sheath (*bs*); nucleus of the bacteriocyte (*white arrow*). LM, scale bar = 20 μm. **e** Fragment of the bacteriocyte with bacteria *Sulcia* (*bcS*). Bacterium *Sulcia* (*S*). TEM, scale bar = 2 μm. **f** Fragment of the bacteriome with *Sodalis*-like bacteria (*bmSd*). Bacteriocyte nucleus (*white arrow*). LM, scale bar = 20 μm. **g** Fragment of the bacteriocyte with *Sodalis-*like bacteria (*bcSd*). Note the small, rod-shaped bacteria in the cytoplasm of the bacteriocyte (*white arrowheads*). Bacterium *Sodalis* (*Sd*). TEM, scale bar = 2 μm. **h** Rectal organ in the lumen of the hindgut. Hindgut (*h*); rectal organ (*ro*); lumen of the hindgut (*black asterisk*). LM, scale bar = 20 μm. **i** A fragment of the rectal organ. Bacteria occupying the rectal organ (*rb*). TEM, scale bar = 2 μm. LM, scale bar = 20 μm. **j** FISH detection of the *Vidania* symbiont in the rectal organ. Hindgut (*h*); rectal organ (*ro*). Confocal microscope, scale bar = 20 μm

Histological observations have shown that bacteria associated with *O. dissimilis* are located both in the bacteriocytes as well as in the fat body cells and other tissue. The analysis of serial semithin sections has revealed the presence of three types of bacteriomes in the abdomen of the insects examined (Fig. 3a). Two of them are tube-shaped, whereas the third one is ovoid in shape. Each of the bacteriomes contains one type of symbiont (Fig. 3a–g). The tube-shaped bacteriomes are surrounded by a single layer of epithelial cells (i.e., bacteriome sheath) (Fig. 3a, d). These tube-shaped bacteriomes are filled with extremely large, lobated bacteria (Fig. 3a–c), as well as with pleomorphic bacteria, respectively (Fig. 3d, e). Pleomorphic bacteria stain intensively with methylene blue (Fig. 3d). In the cytoplasm of these bacteria, single, large, electron-dense inclusions are present (Fig. 3e). In contrast to the pleomorphic bacteria, the large, lobated ones stain less intensively with methylene blue (Fig. 3b) and are characterized by numerous electron-dense inclusions in the cytoplasm (Fig. 3c). The lobated bacteria adhere closely to one another (Fig. 3a, b). The ovoid bacteriomes contain bacteriocytes with large, elongated bacteria (Fig. 3f), which are electron-translucent under TEM (Fig. 3g). These bacteriomes do not possess a bacteriome sheath. The bacteriomes containing the pleomorphic and elongated bacteria are made up of several large bacteriocytes, whereas the bacteriomes with lobated bacteria are syncytial. Both bacteriocytes and syncytium possess giant nuclei (Fig. 3b, f) and cytoplasm tightly packed with bacteria, ribosomes, and mitochondria (Fig. 3c, e, g).

**Fig. 4:**
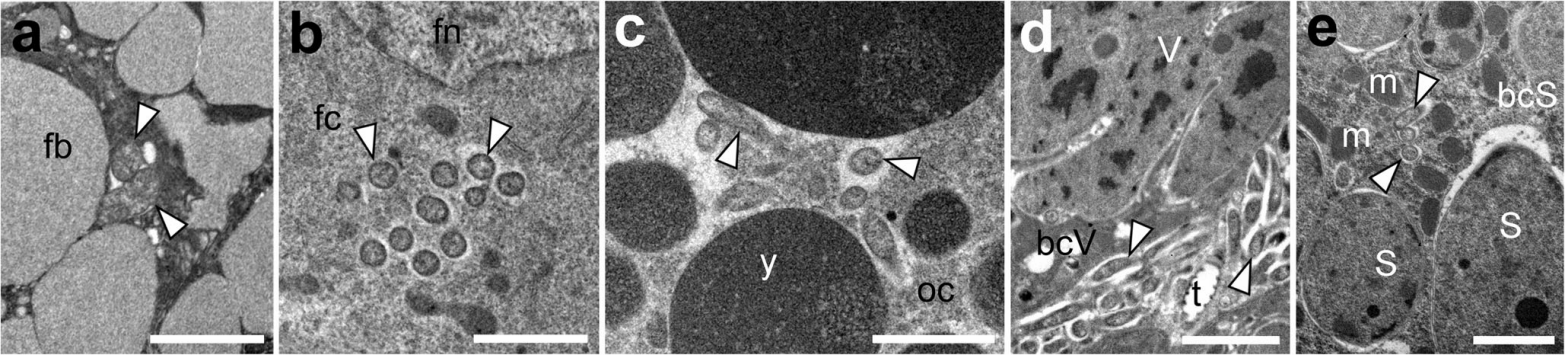
Distribution of small rod-shaped bacteria (*Wolbachia/Rickettsia*) in the body of *O. dissimilis*. **a** Bacteria (*white arrowheads*) in the fat body cells (*fb*), TEM, scale bar = 2 μm. **b** Bacteria (*white arrowheads*) in the cytoplasm of the follicular cell (*fc*). Follicular cell nucleus (*fn*). TEM, scale bar = 2 μm. **c** Bacteria (*white arrowheads*) in the cytoplasm of the oocyte (*oc*). Yolk granule (*y*). TEM, scale bar = 2 μm. **d** Bacteria (*white arrowheads*) in the cytoplasm of the bacteriocyte with bacteria *Vidania* (*bcV*). Trachea (*t*); bacterium *Vidania* (*V*). TEM, scale bar = 2 μm. **e** Bacteria (*white arrowheads*) in the cytoplasm of the bacteriocyte with bacteria *Sulcia* (*bcS*). Mitochondrium (*m*); bacterium *Sulcia* (*S*). TEM, scale bar = 2 μm

Apart from bacteriocyte-associated symbionts, small, rod-shaped bacteria were observed in *O. dissimilis* (Figs. 3g and 4a–e). These bacteria are dispersed throughout the different insect tissue, such as fat body cells, follicular cells, and oocytes (Fig. 4a–c). They also occur in the cytoplasm of all the bacteriocytes (Figs. 3g and 4d, e). Taking into account their shape and size, it seems that these microorganisms may represent the bacteria *Wolbachia* and *Rickettsia* detected in molecular cloning, however based on current data their detailed identification is impossible.

**Fig. 5:**
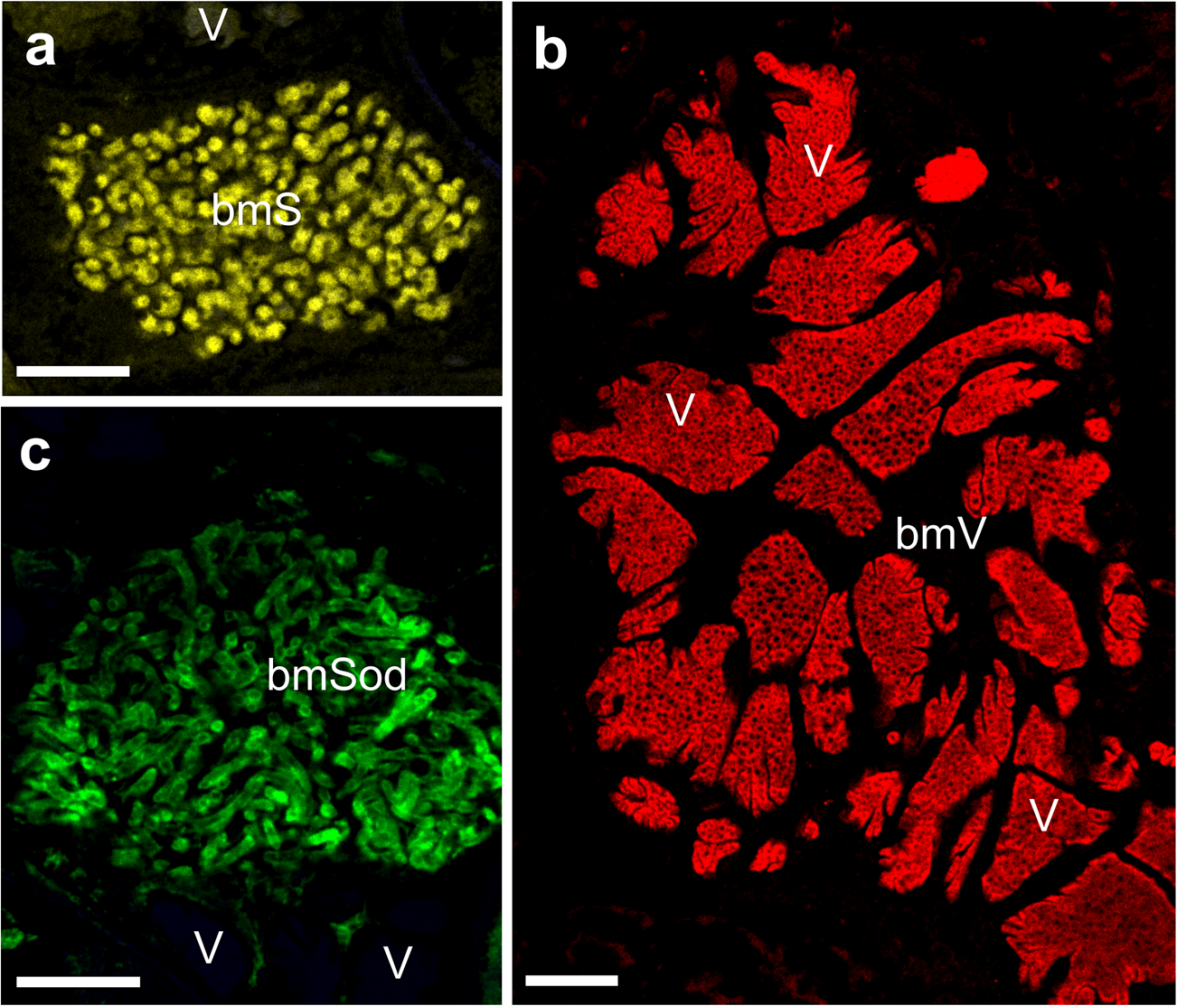
In situ identification of symbionts of *Ommatidiotus dissimilis*. Symbiont-specific 16S rRNA targeted probes were labeled with Cy-3, Cy-5, and FITC for the *Sodalis*, *Vidania*, and *Sulcia* symbionts, respectively. **a** Bacteriocyte filled with bacteria *Sulcia* (*bmS*). Bacterium *Vidania* (*V*). Confocal microscope, scale bar = 20 μm. **b** Two neighboring bacteriocytes (*bmV*) with bacteria *Vidania* (*V*). Confocal microscope, scale bar = 20 μm. **c** Fragment of the bacteriocyte (*bmSod*) with rod-shaped bacteria belonging to the genus *Sodalis*. Bacterium *Vidania* (*V*). Confocal microscope, scale bar = 20 μm

The bacteriocyte-associated symbiotic bacteria of *O. dissimilis* were identified through the combination of results of histological, ultrastructural, and molecular analyses. Based on the characteristic structural and ultrastructural features of the symbionts residing in auchenorrhynchous Hemiptera (Bressan et al. 2009, 2013; Noda et al. 2012; Brentassi et al. 2017; Michalik et al. 2014; Kobiałka et al. 2015, 2016, 2017; Szklarzewicz et al. 2016) as well as results of the fluorescence in situ hybridization with symbiont-specific probes (Fig. 5a–c), the pleomorphic bacteria have been identified as *Sulcia*, the large, lobated ones as *Vidania*, and the large, elongated ones have been identified as *Sodalis*-like bacteria.

Apart from bacteriomes which occur in the body cavity, *O. dissimilis* also possesses an additional, single bacteriome (which Buchner calls the “rectal organ”) localized in the lumen of the hindgut (Fig. 3h–j). The analysis of semithin serial sections has shown that this bacteriome is composed of several bacteriocytes filled with one type of small, pleomorphic bacteria (Fig. 3h–j). The FISH assay has revealed that bacteria occupying the bacteriome in the lumen of the hindgut represent bacteria *Vidania* (Fig. 3j).

### Symbionts of *Ommatidiotus dissimilis* are transovarially transmitted between generations

**Fig. 6:**
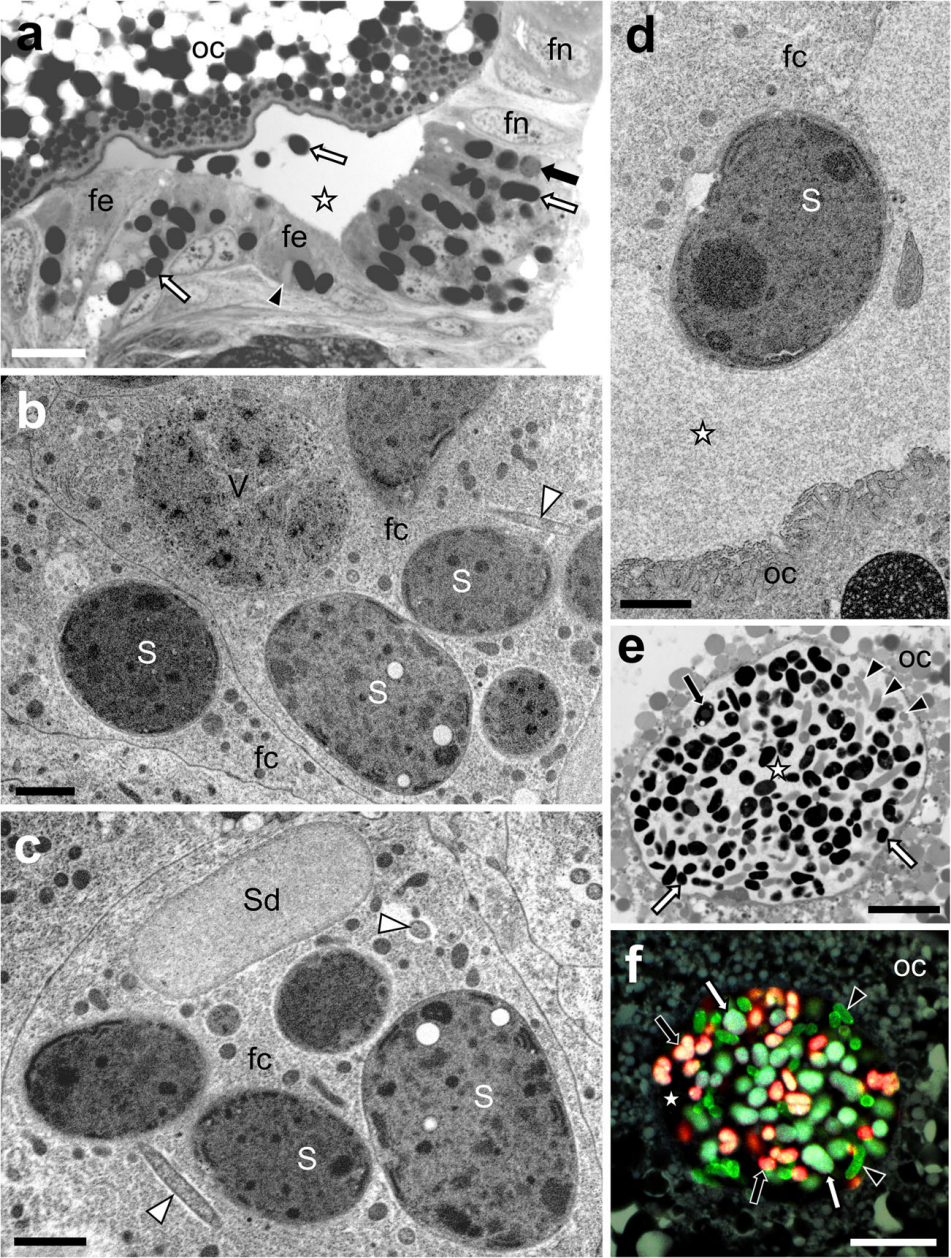
Consecutive stages of transovarial transmission of symbiotic bacteria in *Ommatidiotus dissimilis*. **a** The migration of the symbiotic bacteria through the follicular epithelium surrounding the posterior pole of the terminal oocyte to the previtelline space. Bacterium *Sulcia* (*white arrow*); bacterium *Vidania* (*black arrow*); *Sodalis-like* bacterium (*black arrowhead*); follicular epithelium (*fe*); nucleus of the follicular cell (*fn*); oocyte (*oc*); perivitelline space (*white asterisk*). LM, scale bar = 20 μm. **b**, **c** Symbiotic bacteria in the cytoplasm of the follicular cells (*fc*). Bacterium *Sulcia* (*S*); bacterium *Vidania* (*V*); *Sodalis*-like bacterium (*Sd*); small, rod-shaped bacterium (*white arrowhead*). TEM, scale bar = 2 μm. **d** Bacterium *Sulcia* (*S*) migrating from the follicular cell to the perivitelline space. Follicular cell (*fc*); oocyte (*oc*); perivitelline space (*white asterisk*). TEM. Scale bar = 2 μm. **e** A “symbiont ball” in the deep depression of the oolemma. Bacterium *Sulcia* (*white arrow*); bacterium *Vidania* (*black arrow*); *Sodalis-like* bacterium (*black arrowhead*); oocyte (*oc*); perivitelline space (*white asterisk*). LM, scale bar = 20 μm. **f** FISH detection of bacteria *Sulcia* (*white arrow*), *Vidania* (*black arrow*), and *Sodalis* (*black arrowhead*) in the “symbiont ball.” Oocyte (*oc*); perivitelline space (*white asterisk*). Confocal microscope, scale bar = 20 μm

Analyses of semithin sections have shown that all of the bacteria associated with the planthopper *O. dissimilis* are transovarially (i.e., via female germ cells) transmitted from one generation to the next. The migration of symbionts correlates with the course of oogenesis (for further details concerning organization of ovary and process of oogenesis in Fulgoromorpha, see Szklarzewicz et al. 2007). The beginning of infection takes place at the time the terminal oocytes are in the late vitellogenic stage (Fig. 6a). At this time, the bacteria leave the cytoplasm of the bacteriocytes/syncytial bacteriomes and migrate towards the ovaries. All types of symbionts simultaneously infect the ovarioles (Fig. 6a–c). It was observed that bacteria *Sulcia* and *Vidania* transform before migration. Bacteria *Sulcia* which begin to escape from the bacteriocytes stain more intensely with methylene blue. In turn, *Vidania* symbionts change shape and become almost spherical (Fig. 6a, b). In contrast to *Sulcia* and *Vidania*, the remaining symbionts, i.e., *Wolbachia*, *Rickettsia*, and *Sodalis-*like bacteria, do not change shape during migration (Fig. 6a, b). Symbiotic microorganisms migrate to the perivitelline space through the cytoplasm of follicular cells (Fig. 6a–d). After passing the follicular epithelium, symbionts gather in the perivitelline space, where they create the structure termed the “symbiont ball” (Fig. 6e, f).

The PCR reactions with symbiont-specific primers using a DNA template isolated from fully grown oocytes revealed the presence of all the mentioned above types of bacteria.

## Discussion

Heritable bacterial symbionts are extremely prevalent in Hemiptera and other insects. They are highly diverse and able to form mutualistic relationships with their hosts; they play an important role and have a great impact on various biological functions of their insect partners (Douglas 2014, 2016; Weinert et al. 2015). It is estimated that up to 20% of all insects engage in symbiosis with microbial companions, and it is likely that their capacity in such relationships contributes greatly to their evolutionary success (Baumann 2005; Douglas 1989; Ishikawa 1989; Moran and Baumann 2000; Feldhaar and Gross 2009; Kikuchi 2009; Wernegreen 2012). The past decade has seen an explosion of studies which characterize the biology of hemipteran symbionts, but too little attention has been given to the trophic relationships and ecology of their hosts. *Ommatidiotus dissimilis*, being the object of this study, is a monophagous phloem feeder of *Eriophorum vaginatum*, and inhabits a distinct habitat of acidic peat bogs. Our results have revealed that *O. dissimilis* serves as host to three bacteriocyte-associated, symbiotic bacteria which belong to the genera *Sulcia*, *Vidania*, and *Sodalis*. All of the symbionts mentioned above are localized in separate bacteriomes and are present in all of the individuals investigated. In addition, two types of bacteria representing the genera *Wolbachia* and *Rickettsia* were found. The role of the latter in the biology of the host insect remains unclear, however taking into account the facts that (1) they are transovarially inherited and (2) they do not have a negative impact on the growth and development of the host insect, it may be suggested that *Wolbachia* and *Rickettsia* represent facultative symbionts of *O. dissimilis.* It should be stressed that the case of pentasymbiotic association was reported in Fulgoromorpha only once before, in an unidentified representative of the family Derbidae (or Cixiidae?) (Müller 1940a, b).

There are only a few reports on symbionts harbored in other representatives of Caliscelidae. Trisymbiotic association was reported by Müller (1940a, b) in *Caliscelis bonelli* (Caliscelidae: Caliscelinae: Caliscelini). More recently, Urban and Cryan (2012) molecularly investigated *Aphelonema* sp. (Caliscelidae: Caliscelinae: Peltonotellini), but, surprisingly, they did not detect *Vidania* nor *Sulcia* symbionts in this species. *Caliscelis bonelli* is a Mediterranean, xerothermophilous species, which probably feeds on various Poaceae (Holzinger et al. 2003). The species of the genus *Aphelonema* are distributed in the Nearctic, with feeding records on Poaceae—grasses and sedges, e.g., *Spartina* spp., *Carex* spp*.*, often in marshy or salty areas (Bartlett et al. 2014; Bartlett 2016). Having this limited set of data available, we can hypothesize that the phenomenon of symbiont loss and replacement took place multiple times during the evolution of the Caliscelidae family, most likely as the result of environmental and host plant shifts. The pentasymbiotic association of *O. dissimilis* could be a striking example of the expansion of microbial companions, as three different symbiotic bacteria are present in the bacteriocytes—*Sulcia*, *Vidania*, and *Sodalis*-like bacteria—a combination not found among any other planthoppers.

It should be underlined that this is the first report on the occurrence of a *Sodalis*-like symbiont in Fulgoromorpha. So far, the presence of *Sodalis*-allied bacteria has been reported in several insect groups which feed on different food such as follows: leafhoppers (Koga et al. 2013; Michalik et al. 2014), scale insects (von Dohlen et al. 2001; Husnik and McCutcheon 2016; Szklarzewicz et al. 2018), tsetse flies (Dale and Mauldin 1999), Hippoboscidae flies (Dale and Maudlin 1999; Nováková and Hypša 2007; Chrudimský et al. 2012), Philopteridae lice (Fukatsu et al. 2007), weevils (Toju and Fukatsu 2011), and some stinkbugs (Kaiwa et al. 2010). The relationships between insects and *Sodalis*-like bacteria are usually considered relatively recent due to the fact that they possess relatively large genomes, which are larger than the genomes of long-established symbionts and are comparable to the genome size of free-living bacteria (Husnik and McCutcheon 2016). The symbiosis between insects and bacteria belonging to the genus *Sodalis* usually is a result of the symbiont replacement process. For example, in the grain weevils belonging to the genus *Sitophilus*, *Sodalis*-allied bacteria probably replaced their ancient symbiont *Nardonella* (Conord et al. 2008; Toju et al. 2013). The phenomenon of the replacement of ancient symbionts by *Sodalis*-like bacteria has also been reported in some spittlebugs (Koga et al. 2013). Recent molecular studies conducted by Koga et al. (2013) have indicated that in the family of Aphrophoridae (subfamily Aphrophorinae; tribe Philaenini), the ancestral symbiont—betaproteobacterium *Zinderia* has been replaced by *Sodalis*-like bacterium; however, Philaenini probably acquired these bacteria in multiple, independent infections. Symbiosis “*in statu nascendi*” between insect and *Sodalis*-allied bacteria was also observed in the green leafhopper *Cicadella viridis*, in which *Sodalis*-like bacterium most likely substituted the typical of the majority of Cicadellidae *Baumannia* symbiont (Michalik et al. 2014). It should be added that the results of our molecular analyses of symbionts in *O. dissimilis* agree with the hypothesis of the relatively recent origin of *Sodalis*-insects interactions. BLAST searches have indicated that 16S RNA gene sequences of *Sodalis*-like symbiont of *O. dissimilis* exhibit a high similarity (98% identity) to the homologous sequence of the free-living bacterium, *Sodalis praecaptivus.* What is also interesting is that in a natural environment, *Sodalis praecaptivus* may occur both in plant and animal tissue; therefore, taking into account the above-mentioned facts, it may be suggested that *S. praecaptivus* represents the ancestor of *Sodalis*-like symbionts of insects.

The function of *Sodalis*-like bacteria in *O. dissimilis* remains unknown, but it seems probable that it may be representative of a third obligate symbiont, which complements *Sulcia* and *Vidania* with respect to the production of essential nutrients to the host insect. The observed situation may also be an intermediate stage of symbiosis, in which *Sodalis*-like bacteria represent recently acquired symbionts, which could possibly replace the *Sulcia* or *Vidania* symbiont in the future. However, to determine whether the *Sodalis*-like symbiont only supplements the lacking genes of residing symbionts or the residing symbionts lost genes occurring in the genome of *Sodalis*-like bacteria, more detailed molecular investigations including that of genome sequencing are needed.

Two of the additional bacteria found in *O. dissimilis* were *Wolbachia* and *Rickettsia*. *Wolbachia* is believed to infect a large number of insect species worldwide (Zug and Hammerstein 2012). In our sample of *O. dissimilis*, the prevalence of *Wolbachia* was very high and all of the examined specimens were infected; the bacteria were detected in molecular investigations and also observed under electron transmission microscope. The discovery of *Rickettsia* as the fifth symbiont of *O. dissimilis* was confirmed using molecular techniques. It belongs to the *bellii* group and its 16S RNA gene sequence concordant follows in 99% these of *Rickettsia* from the green leafhopper *Cicadella viridis* and tick *Dermacentror variablis*. It is estimated that the *belli* group of *Rickettsia* split about 50 million years ago, and their primary hosts in this group were, exclusively, arthropods (Weinert et al. 2009a, b; Weinert 2015). The exact mechanism of transmission of this bacterium between hemipterans has not been recognized; however, *Rickettsia* is thought to be horizontally transmitted through plants (Caspi-Fluger et al. 2012; Weinert 2015).

To date, very little is known about the function of the rectal organ in Fulgoromorpha. Müller (1940a, b, 1961) and Buchner (1965) suggested that the rectum of the females of Fulgoromorpha is occupied by *x*-symbionts (i.e., bacterium *Vidania*). Our results of fluorescence hybridization in situ confirmed that the symbionts residing within the rectal organ of *O. dissimilis* represent the bacterium *Vidania*. Our findings are in agreement with earlier observations of the rectal organ of the planthopper *Oliarus filicicola* (Cixiidae), conducted by Bressan and Mulligan (2013). On account of a complete lack of data on the function of the rectal organ, its role remains unclear. Buchner (1965) suggested that this structure serves for the transmission of *x*-symbionts to the progeny. Buchner based this supposition on the fact that he did not observe *x*-symbionts during egg infection. Since our results have revealed that the bacterium *Vidania* (i.e., *x*-symbiont sensu Buchner and Müller) is transovarially shifted between generations (see below), the function of the rectal organ requires further comprehensive study.

So far, the data concerning the modes of symbiont inheritance in Fulgoromorpha are merely fragmentary (Müller 1940a, b; Buchner 1965; Cheng and Hou 2001; Szklarzewicz et al. 2007; Michalik et al. 2009; Yukuhiro et al. 2014). Buchner (1965) and Müller (1940a, b) described the transfer of symbionts in some fulgoroids using histological methods. They showed that symbiotic bacteria of Fulgoromorpha might be transferred from mother to offspring in various ways. They may infect undifferentiated germ cells or fully grown oocytes. Additionally, Buchner (1965) observed that in trisymbiotic planthoppers, one type of symbiont infects the cystocytes (i.e., undifferentiated germ cells) and is then transmitted to the oocyte via the nutritive cord, whereas the remaining two invade the posterior pole of fully grown oocytes. This observation has been confirmed by Szklarzewicz et al. (2007), who conducted an analysis of the ovaries of *Cixius nervosus* (Cixiidae) by means of ultrastructural methods. This paper provides the first description of bacterial symbiont transmission in Fulgoromorpha on an ultrastructural level. Our observations have indicated that in *O. dissimilis* all five types of obligate bacterial symbionts infect the ovarioles simultaneously in the same manner. They migrate to the perivitelline space through the follicular epithelium, which surrounds the posterior pole of the terminal oocyte. It is especially interesting that despite their extremely large size bacteria, *Vidania* also migrate through the cytoplasm of follicular cells. It is worth noting that Buchner did not observe the bacteria corresponding to *Vidania* symbiont during the oocyte infection (see above), and he speculated that these symbionts are not transovarially transmitted between generations. Although it seems very probable that due to the fact that the bacterium *Vidania* changes shape before leaving the bacteriocyte, and becomes almost spherical (and smaller than in bacteriocytes) during the migration to the ovaries, he did not recognize this bacterium under a light microscope. Furthermore, the molecular studies conducted by Urban and Cryan (2012) revealed the co-evolution between *Vidania* and its planthopper hosts, which supports our observation on their transovarial transmission preventing the horizontal gene transfer between symbionts and free-living bacteria.

The host plant of *O. dissimilis*—the hare’s-tail cottongrass *Eriophorum vaginatum*—has a highly developed tolerance to low resource availability, as it resides and flourishes in cold and infertile sites and is capable of growing over a large range of moist conditions (Silvan et al. 2005; Mauqoy and van Geel 2013). The properties of the plant, its habitat and evolutionary history (Spalink et al. 2016; Waterway et al. 2016) have led us to the hypothesis that the associations with additional microbial companions of *O. dissimilis* are relatively recent, probably resulting from niche differentiation during the past few million years, especially during the dynamic glacial cycles of the Pleistocene which affect both of the host plants and planthoppers that feed on them.

In summary, the results of our morphological, ultrastructural, and molecular analyses of the symbiotic system of *O. dissimilis* provide evidence for a large diversity of symbionts in Fulgoromorpha: not only did they confirm the presence of ancestral symbionts, i.e., bacteria *Sulcia* and *Vidania*, but they also revealed the occurrence of *Sodalis*—like symbionts in Fulgoromorpha for the first time. To our knowledge, we are the first to report the transovarial transmission of bacteria *Vidania* between generations. The results obtained revealed the great diversity of symbionts of Fulgoromorpha; however, further analyses of symbionts of other members of Fulgoromorpha are needed in order to better understand the nature of symbioses in this insect group.
